# Dose Verification in Intensity Modulation Radiation Therapy: A Fractal Dimension Characteristics Study

**DOI:** 10.1155/2013/349437

**Published:** 2013-07-08

**Authors:** Jia-Ming Wu, Chung-Ming Kuo, Ching-Jiang Chen

**Affiliations:** ^1^Department of Information Engineering, I-Shou University, No. 1, Sec. 1, Syuecheng Road, Dashu District, Kaohsiung City 84001, Taiwan; ^2^Department of Radiation Oncology, E-Da Hospital, Kaohsiung, Taiwan; ^3^Department of medical Imaging and Radiological Science, Central Taiwan University of Science and Technology, Taichung, Taiwan; ^4^Department of Medical Imaging and Radiological Sciences, I-Shou University, No. 1, Sec. 1, Syuecheng Road, Dashu District, Kaohsiung City 84001, Taiwan

## Abstract

*Purpose.* This study describes how to identify the coincidence of desired planning isodose curves with film experimental results by using a mathematical fractal dimension characteristic method to avoid the errors caused by visual inspection in the intensity modulation radiation therapy (IMRT). *Methods and Materials.* The isodose curves of the films delivered by linear accelerator according to Plato treatment planning system were acquired using Osiris software to aim directly at a single interested dose curve for fractal characteristic analysis. The results were compared with the corresponding planning desired isodose curves for fractal dimension analysis in order to determine the acceptable confidence level between the planning and the measurement. *Results.* The film measured isodose curves and computer planning curves were deemed identical in dose distribution if their fractal dimensions are within some criteria which suggested that the fractal dimension is a unique fingerprint of a curve in checking the planning and film measurement results. The dose measured results of the film were presumed to be the same if their fractal dimension was within 1%. *Conclusions.* This quantitative rather than qualitative comparison done by fractal dimension numerical analysis helps to decrease the quality assurance errors in IMRT dosimetry verification.

## 1. Introduction

Cancer has been treated by using radiation for more than a century, and, today, more than half of all cancer treatments utilize radiation therapy. Intensity modulated radiation therapy (IMRT) [1–6] is a remarkably advanced radiation therapy technique for the treatment of various kinds of cancers. The computer then optimizes the best treatment to maximize the radiation dose delivered to the tumor while minimizing the radiation dose delivered to the surrounding normal tissues [7–9]. However, not only the planning of treatment but also the dose delivery technique is more complicated than for three dimensional conformal therapy [10]. In other words, the importance of quality assurance (QA) [11] procedure in Intensity Modulation Radiation Therapy (IMRT) should be enhanced compared to that of conventional conformal radiation treatment. 

The QA process usually includes verification of intensity map to radiation field coincidence by film [12]. Irrespective of the method chosen for quality assurance, dosimetric verification criteria for IMRT treatment plans are based upon either the analysis of a limited number of points in low-dose gradient areas or the measurement of distances between isodose lines in high-dose gradient areas. Radiation oncologists and medical physicists usually compare the desired dose and film measurement results by placing these transparency films side by side to visualize their discrepancy or by superimposing these films of isodose curves onto planning results to check the difference. Whatever method is used, visual inspection leads to in accordance with person to person philosophical errors. The integrity of complexity of the IMRT dose delivery technique relies on quantification of the coincidence of the planned and delivered intensity-modulated radiation therapy dose distributions. 

The aim of this study was to ascertain how to identify the coincidence of the planned and desired isodose curves and experimental film results, without visual inspection but using a mathematical method to estimate the error between the planned and measured values.

## 2. Materials and Methods

The treatment planning system Plato was used to implement IMRT for cancer treatment and the Elekta precise linear accelerator “step and shoot” technique was used to deliver the planned desired dose. The output was first checked before IMRT QA; this was normally performed for a standard set. Relative dosimetry was given to all subsequent measurements, which were compared to the dose at the absolute calibration point. It was not practical to check the patient dose by imitating the patient contour and anatomy case by case. Therefore, before the treatment plan was accomplished, the planning parameters were acquired from a cubic solid water phantom, from which images were acquired in advance of implementing a test planning. This was done by setting the irradiation beam onto the cubic phantom surface vertically according to the patient planning parameters portal by portal to simplify the dose distribution checking procedure. 

The dose distributions adopted in the pseudocubic phantom were delivered by using a linear accelerator, and the irradiation fields were measured with a therapy verification film (Kodak, X-Omat V, Eastman Kodak Company, Rochester, NY, USA) using a standard procedure. The film was placed in a solid water phantom (PTW, white polystyrene “RW3,” PTWFREIBURG, Freiburg, Germany) and developed by means of an automatic procedure. No specific calibration was made; however, the film was exposed to a dose value to guarantee that it was in the dose-density linear region of the H-D curve [13]. All films were read with an optical scanner (Vidar, VXR-12, VIDAR Systems Corporation, Herndon, VA, USA) to create the relative isodose curves. These isodose curves were acquired using Osiris (Geneva University Hospital, version 3.5) to aim directly at a single interested dose curve for fractal [14] characteristic analysis, described in detail later. All dose curves were composed of the segments, and each segment was composed of beamlets. The combination of beamlets penumbra and superimposition of tiny open fields during dose delivery led to each isodose curve having its own exclusive fractal characteristics. One of the interesting curves was selected to measure the length or area encompassed by the curve. The fundamental idea is to assume that the two quantities—the length of the curve and the scale—do not vary arbitrarily but instead are related by a law which allows us to compute one quantity from the other. 

### 2.1. Self-Similarity Dimension

**Figure 1:**
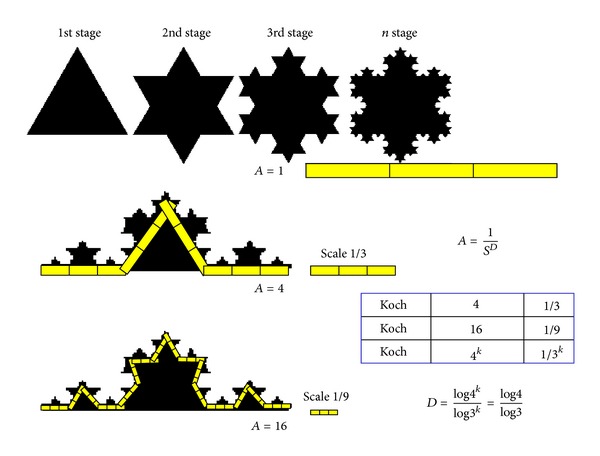
This figure illustrates the understanding and meaning of the power law behavior in a pure mathematical situation in Koch Island. Each Koch curve can be divided into four self-similar parts, which are similar to the entire curve via a similarity transformation which in turn is similar to the entire curve of Figure 3.

According to Figure 1, the Koch curve can be divided into four self-similar parts, which are similar to the entire curve via a similarity transformation which is reduced by a reduction factor of 3, with the relationship *A* = 1/*S* ^Ds^ (where *A* represents the number of bar pieces, Ds denotes the self-similarity dimension, and *S* is the scale of reduction factor). Similarly, for the interested line, there is a nice power law relationship between the length of bar pieces *u* and the reduction factor *S*. This law is *u* = 1/*S* ^*d*^, where *d* is the slope in the log/log diagram. Here, we can introduce the relationship between the power law of the length measurement using different compass settings and the self-similarity dimension of a fractal curve, and use this self-similarity dimension as the identification of a curve. The relationship is simple, namely, Ds = 1 + *d*, where *d* denotes the slope in the log⁡/log⁡ diagram of the length of bar pieces *u* versus precision 1/*S*; that is, *u* = *c*/*S* ^*d*^, and we simplify by choosing appropriate units of length measurements such that the factor *c* in the power law becomes unity, and *u* = 1/*S* ^*d*^. 

Taking into consideration logarithms, we obtain

(a) $$\begin{array}{c} \log u=d\cdot \log\frac{1}{S}, \end{array}$$

where, again, *u* is the length of bar pieces with respect to compass settings. On the other hand, we have the power law *A* = 1/*S* ^Ds^, where *A* denotes the number of bar pieces in a replacement step of the self-similar fractal with scale reduction factor *S*. In the logarithmic form, this is

(b) $$\begin{array}{c} \log A=\text{Ds}\cdot \log\frac{1}{S}. \end{array}$$

Note that the connection between *u* (the length of bar) and *A* stands for the number of pieces, when measuring at some other scales, where the whole object is composed of a small copies each of size *S*, and then we measure a total length of *A* times *S*, and  *u* = *A* · *S*. Taking logarithms into consideration again,

(1) $$\begin{array}{c} \log u=\log A+\log S. \end{array}$$

In this equation, we can substitute the logarithms log⁡⁡*u* and log⁡⁡*A* from ((a)) and ((b)) above.

This yields

(2) $$\begin{array}{c} d\cdot \log\frac{1}{S}=\text{Ds}\cdot \log\frac{1}{S}+\log S. \end{array}$$

Since

(3) $$\begin{array}{c} \log\frac{1}{S}=-\log S, \end{array}$$

we get

(4) $$\begin{array}{c} -d\cdot \log S=-\text{Ds}\cdot \log S+\log S. \end{array}$$

Dividing by log⁡⁡*S* and sorting terms, we finally arrive at

(5) $$\begin{array}{c} \text{Ds}=1+d. \end{array}$$

### 2.2. Reduction Factor of Geometry Structure

**Figure 2:**
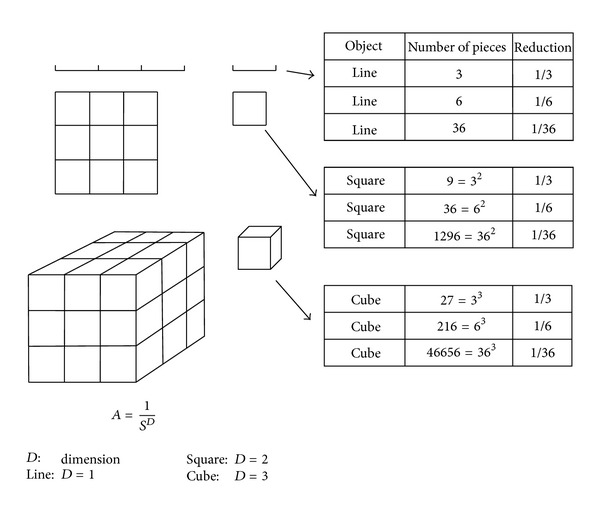
The relationship between the reduction factor (scaling factor) and the number of scaleddown pieces into which the structure is divided. Apparently, for the line, square, and cube, there is a nice power law relationship between the numbers of pieces, *a*, and the reduction factors. This law is *a* = 1/*S* ^*D*^ where *D* = 1 for the line, *D* = 2 for the square, and *D* = 3 for the cube.

According to Figure 2 and the relationship between the reduction factor (scaling factor) and the number of scaled down pieces into which the structure is divided, apparently, for the line, square, and cube, there is a nice power law relationship between the numbers of pieces and the reduction factors. This is the law relationship between the number of pieces and the reduction factors. This law is

(6) $$\begin{array}{c} A=\frac{1}{S^{D}}, \end{array}$$

where *D* = 1 for the line, *D* = 2 for the square, and *D* = 3 for the cube. Here we see that the reduction factor is 1/3 which is, of course, arbitrary. We could alternatively have chosen 1/2, 1/7, or 1/365. However, precisely in this fact lies the difference between these figures and the isodose curves in which we are interested (fractal structures). The reduction factors are characteristic for any fractal structures, so that the self-similarity dimension is a unique choice to represent an isodose curve.

### 2.3. Film Manipulation after Dose Delivery

**Figure 3:**
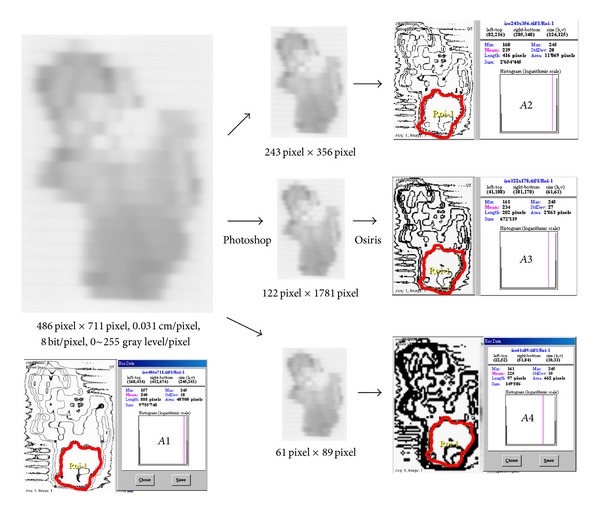
This figure shows how the dose curve is adopted for fractal analysis. The original resolution (left on the figure) is 486 pixels × 711 pixels with 8 bits/pixel. The scale is then reduced by 1/2, 1/4, and 1/8 (from right up to right bottom) to give 243 × 356, 122 × 178, and 61 × 89 pixels to measure the length of the same isodose curve adopted in the film. The planning dose curve Ds is then compared to that of planning result. The curve of low resolution (lower right) is coarse and big while high resolution (upper right) shows slim curves.

In Figure 3, film images are acquired using Osiris from FIPS Laser densitometer at a resolution of 486  ×  711 pixels with 8 bits/pixel. Consider a region of interest (e.g., an isodose curve in which we are interested), then measure the length of this curve, and reduce to the original scales of 1/2, 1/4, 1/8, which are 243  ×  356, 122  ×  178, and 61  ×  89 pixels, respectively. It is obviously the low resolution (lower right) which reveals coarse and big curves while slim curves are shown for high resolution (upper right).

### 2.4. Length versus (1  *⁄*  Scale) Logarithms

**Figure 4:**
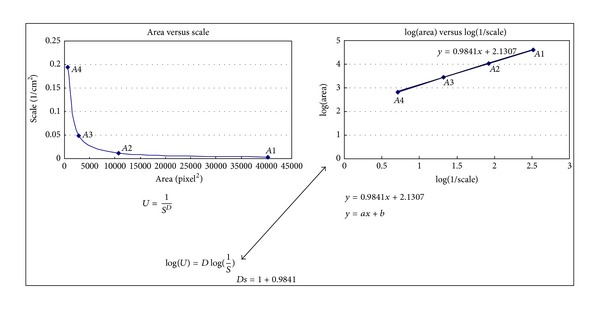
The law seems to be relevant of scale verse tarea on left hand side in this figure, which is a power law of the form *y* ∝ *x* ^*d*^ (where *y* denotes the length, *x* denote the scale, and d is the dimension). Take the logarithm of the length of each region of interest and logarithm (1/scale), and then plot log⁡⁡(length) against log⁡⁡(1/scale); then the slope is in the form *y* = *ax* + *b*. The slope can represent the unique characteristics of the curve.

In Figure 4, take the logarithm of the length of each region of interest and logarithm (1/scale), and then plot the log⁡⁡(length) against log⁡⁡(1/scale), where the slope is in the form *y* = *ax* + *b*. According to Figure 4, the slope of the fitted line is 0.9841 and, in accordance, Ds = 1 + *d*; that is, the self-similarity dimension is equal to 1 + 0.9841.

### 2.5. Plan Isodose Curves Manipulation

The plan isodose curves are also acquired using Osiris for deriving their self-similarity dimension. The original format is 486 × 711 pixels with 8 bits/pixel; the scale is then reduced by 1/2, 1/4 and 1/8, which gives 243 × 356, 122 × 178 and 61 × 89 pixels to measure the length of the same isodose curve adopted in the film. The planning dose curves, Ds, are then compared to those of the film result.

## 3. Results

Validation studies carried out in this manner have consistently shown point doses delivered at isocenter using Elekta medical linear accelerator to be within a maximum of 3.5% of those predicted by Plato treatment planning system in high-dose, low-gradient regions, with 99% of points in high-gradient, high-dose regions falling within 3.6 mm of predicted positions. 

Phantom plans and film images were registered and normalized at the cross isocenter for further fractal study. The percentage of the pixels in high-dose low-gradient areas of the dose distribution for all analyzed phantom plans was within the 3.5% tolerance level. The percentage of the pixels throughout the entire area of the dose distribution for all analyzed phantom plans was within the 10% tolerance level. 

Figure 3 shows an interesting phenomenon: dose curves are coarse and big for low resolution while slim curves are found for high resolution. When checking the length or area encompassed by the interested curve, the length or area appears as a geometric progression, followed by an increasing scale rather than an arithmetic progression. 

**Figure 5:**
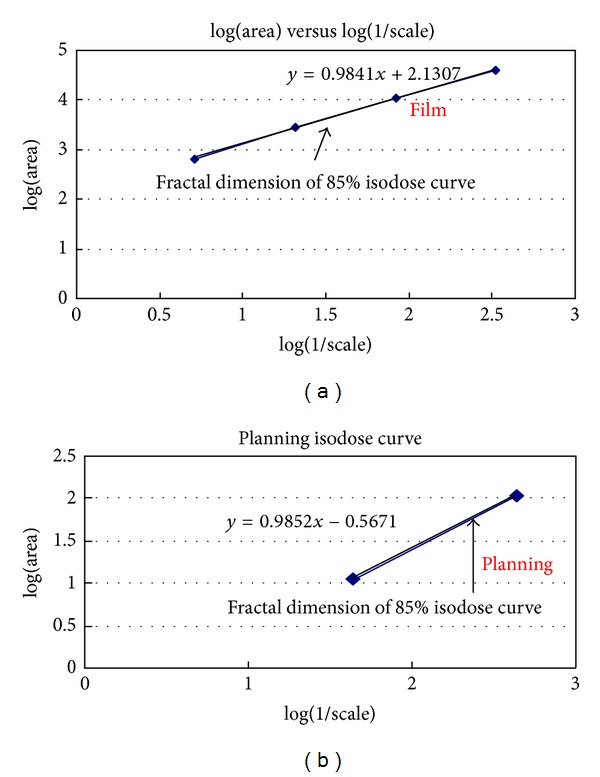
When these two dose distributions are normalized at their cross hair isocenter, the 88% region of the interested planning isodose curve's Ds is 1.9852, and the 88% region of the interested film isodose curve's Ds is 1.9841. The discrepancy is only 5 × 10^−4^[(1.9852 − 1.9841)/1.9852]. The planning 88% isodose curve and film 88% isodose curve are supposed to be identical if the difference between their Ds values is within 1%.

The 85% region of the interested planning isodose curve's Ds is 1.9852, and the 85% region of the interested film isodose curve's Ds is 1.9841. The discrepancy is only 5 × 10^−4^[(1.9852 − 1.9841)/1.9852] (as in Figure 5). The planning 85% isodose curve and film 85% isodose curve are supposed to be identical if the differences between their Ds values are within 1%.

## 4. Discussion

A fractal dimension is a ratio providing a statistical index of complexity comparing how the detail in a pattern (strictly speaking, a fractal pattern) changes with the scale at which it is measured. Consequently, it is necessary to develop sophisticated tools to compare measured and calculated dose distributions in order to verify the accuracy of the results of the planned dose distribution. Different methods have been developed to evaluate the accordance between measured and calculated doses, such as the point-to-point dose difference or the evaluation of the distance between two closed points having the same dose value. The verification method proposed by Low seems to be more complete since it takes into account both the dose difference (DD) and the distance to agreement (DTA), allowing the definition of a “score” of an interested dose distribution. The gamma value test at each point of interest gives real-time information useful for the decision making of the treatment plan. All these methods play different roles in dose verification, and this study describes how to identify the coincidence of desired planning isodose curves with film experimental results by using a mathematical fractal dimension characteristic method to avoid the errors caused by visual inspection.

### 4.1. Mosaic Amalgamation

**Figure 6:**
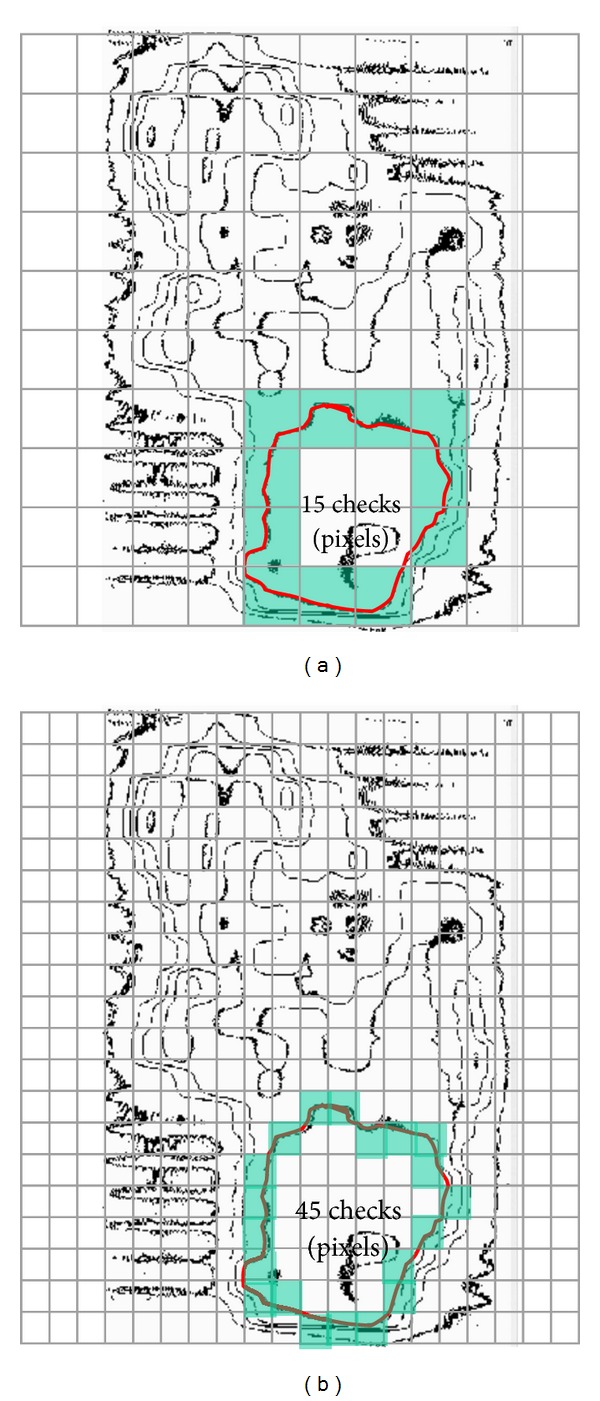
The relationship between length and scale (tiles size) can be implemented by transforming an image into a mosaic and regarding the elements (individual tiles) of the mosaic as being square tiles laid around the boundary. The mosaic transformation can be used to set up a procedure for evaluating the fractal structure of the boundary by a technique known as mosaic amalgamation. The perimeter estimated in (a) is smaller than (b) due to the larger scale (the length of the mosaic tile) used in (a).

When the coincidence of film and fluence map created by IMRT plan is compared, the position of light field borders is normally evaluated by visual observation [15]. It is trivial that this method is subjective, and each operator may introduce an error in locating each border. Strictly, the light field border should coincide with the 50% decrement line of the maximum central lightening, and this limit should be measured with the help of an appropriate device [16]. Because the human eye is not able to detect the 50% light field contour exactly, in this work, a photosensitive diode was employed to test the actual size of the light field selected on a linear accelerator. This ratiocination leads us to compare the plan and film results with unavoidable errors by a traditional method based on human observation. In order to avoid the human visualization inaccuracy with numerical manner, when the gray level films are converted to relative isodose curves, the interested isodose curve's length is measured using mosaic amalgamation. The profile (interested isodose curve) to be evaluated is overlaid by a rectangular grid, as shown in Figures 6(a) and 6(b). The square elements of the grid that sit on the boundary can be regarded as square tiles thrown onto the perimeter of the profile. 

### 4.2. Geometric Progression

Planning dose curves were acquired using the Osiris software for further comparison with the film result described previously. First of all, planning dose curves were normalized to their cross hair isocenter, and the matrix was set to the same as the film. The interesting dose curve of 88% was then adopted for fractal dimension analysis as in Figure 5. The fractal dimension of planning 88% was 1.9852, and the discrepancy was only 5 × 10^−4^ when compared to that of film.

### 4.3. Criterion of Acceptability

**Figure 7:**
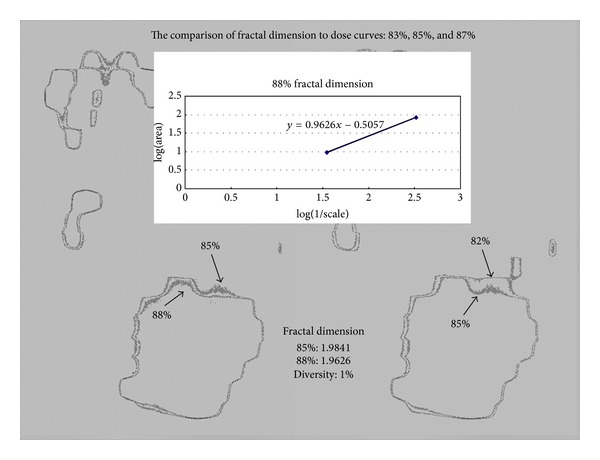
In this figure, the selected 85% is interesting to study. The fractal dimension magnitude criterion of acceptability between the desired planning curves and the delivered dose curves can be made by descending or increasing the isodose curves from 85% downwards or upwards to see which isodose curve is still regarded as one fractal value. When the fractal dimensions, 83% and 87%, are compared to 85% curve, the variation of fractal dimension is within 1%, which means that the two curves are identical only if their fractal dimension is within 1%.

According to Figure 7, the selected curve of 85% is interesting to study. Now the problem arises, when the fractal dimension of planning and films isodose curves are compared, in determining what fractal dimension variation is still acceptable in order to say that the two can be regarded as the same. In order to decide at what range of variation of fractal dimension is still acceptable, we need to check the fractal dimension variation magnitude by varying the dose curves from descending downwards and increasing upwards. If the limitation for the variation in the dose curve was set to be 2%, say 83% and 87%, then the fractal dimension of 85% varied from 1.9841 to 1.9911 and 1.9626 of 83% and 87%, respectively. The variation of fractal dimension is within 1% and the outcomes from the isodose curves descending downwards as well as increasing upwards look acceptable. The results imply that the two curves are identical only if their fractal dimension is within 1%. The fractal dimension provides an easier way to identify the two curves.

### 4.4. Automated Calculation of Fractal Dimension

Work is still ongoing to develop automated calculation of fractal dimension by tracing around the perimeter with an appropriate autosegmentation technique [17]. The coordinates of many points on the perimeter are transferred to the memory to generate data for the evaluation of the fractal structure of the boundary, so that a series of polygons, using a series of paced-out distances along the profile, are constructed.

## 5. Conclusions

Individual IMRT fields generated by the treatment planning system can be verified by film dosimetry in a cubic phantom at a depth of 5 cm. Usually, personnel is used to make side-by-side comparisons of calculated versus measured dose distributions. The calculated and measured dose distributions are compared either superimposed or side by side or by viewing the differences between the two. However, comparing shapes of isodose distributions as measured by film dosimetry and predicted by treatment planning can be more accurately done by numerical analysis as compared to visual inspection. This quantitative rather than qualitative comparison will help decrease errors in dosimetry verification.

Isodose curves measured by film and predicted by computer planning curves are identical if their fractal dimensions are the same. Therefore, fractal dimension is a unique fingerprint of each isodose curve.
